# Autonomic Reactivity to Arousing Stimuli with Social and Non-social Relevance in Alexithymia

**DOI:** 10.3389/fpsyg.2017.00361

**Published:** 2017-03-13

**Authors:** Eduardo S. Martínez-Velázquez, Jacques Honoré, Lucas de Zorzi, Julieta Ramos-Loyo, Henrique Sequeira

**Affiliations:** ^1^Laboratorio de Psicofisiologia, Institute of Neuroscience, University of GuadalajaraGuadalajara, Mexico; ^2^Facultad de Psicología, Meritorious Autonomous University of PueblaPuebla, Mexico; ^3^DEEP Team, SCALab, UMR 9193, CNRS & University of LilleLille, France

**Keywords:** alexithymia, empathy, emotion, IAPS, social relevance, autonomic, skin conductance response, arousal

## Abstract

Emotional difficulties in alexithymia and their social consequences have been linked to alterations in autonomic nervous system. However, most of previous studies did not take into account the distinction between the affective and the cognitive dimensions of the alexithymia, leading to inconsistent results.

**Aim:** In this study, we compared the effects of both dimensions of alexithymia on the autonomic arousal to emotional and social visual stimulations.

**Methods:** Skin conductance responses (SCRs) to items of the International Affective Pictures System characterized by emotional (unpleasant, neutral, and pleasant), social (with humans) or non-social (without humans) content were recorded in non-alexithymic (NA), affective (AA) and cognitive alexithymic (CA) participants, selected on the basis of the Toronto Alexithymia Scale and the Bermond-Vorst Alexithymia Questionnaire. All participants responded to questionnaires of empathy, social phobia, depression, and anxiety before the experiment and evaluated the arousal of the pictures after it.

**Results:** Cognitive alexithymic group showed lower amplitudes of SCRs to pictures with social than without social relevance whereas the opposite pattern was observed for the NA group. Arousal emotional effects of the pictures on SCRs did not differ among groups. In addition, CA participants showed lower scores than NA in the Personal Taking sub-scale of the empathy questionnaire, while AA showed lower scores than NA in the fantasy sub-scale. The CA group showed higher social phobia, depression and anxiety scores, than the other two groups.

**Conclusion:** This work has two original outcomes: first, affective alexithymics expressed lower empathic affective scores than other groups; second, alexithymia modulated the impact of the social relevance of the stimuli on the autonomic reactivity, this impact vanishing in affective alexithymics and reversing in cognitive alexithymics. Thus, though the groups could not be distinguished on the basis of emotional effect on SCRs, they clearly differed when the empathic characteristics and the autonomic impact of social relevance were considered. Finally, the described autonomic signature to social relevant information could contribute to elucidate the difficulty of alexithymics to deal with emotions during social transactions.

## Introduction

Alexithymia corresponds to the difficulty of identifying, analyzing and expressing emotional experiences (Sifneos, 1973; Van der Velde et al., 2013; Goerlich-Dobre et al., 2014). Individuals with alexithymia also present a deficit of imagination and fantasy life, an externally oriented cognitive style (Cochrane et al., 1993; Nandrino et al., 2012) and a difficulty to deal with emotions expressed by others (Taylor et al., 1985). Initially associated with psychosomatic troubles (Nemiah and Sifneos, 1970), alexithymia was later shown to be linked with anxiety (Grynberg et al., 2010, 2012), depression (Hesse and Floyd, 2008; Grynberg et al., 2010), eating disorders (Guilbaud, 2008; Ridout et al., 2010), traumatic experiences (Eichhorn et al., 2014), and autism (Cook et al., 2013). Furthermore, several authors consider alexithymia as a stable personality trait (Mikolajczak and Luminet, 2006) that may occur independently of any clinical condition (Jessimer and Markham, 1997; Vorst and Bermond, 2001; Bermond et al., 2010). Moreover, alexithymic individuals manifest more difficulties (Lane et al., 1996) and a higher latency to recognize facial emotions (Prkachin et al., 2009; Ihme et al., 2014), especially negative ones (Prkachin et al., 2009), as well as low sensitivity to emotional words (Samur et al., 2013). In particular, the ability to recognize emotions in other persons are essential for understanding their intentions and to adapt own behaviors accordingly. Therefore, the difficulties expressed by alexithymics in this ability may be related to emotional dysfunctions in interpersonal and social relationships, or empathy difficulties (Pouga et al., 2010; Bogdanov et al., 2013; Goerlich-Dobre et al., 2015a).

In order to distinguish alexithymia from other close clinical features it appeared necessary, since the emergence of the concept in the seventies, to take into account its dimensions. The Toronto Alexithymia Scale (TAS-20; Bagby et al., 1994), which is now widely used, was designed to assess the difficulty to identify or to describe feelings as well as the presence of externally oriented thinking, in line with the very early description of Nemiah and Sifneos (1970). The more recent Bermond-Vorst Alexithymia Questionnaire (BVAQ; Vorst and Bermond, 2001) brought psychometric evidence that alexithymia could be constituted by two main psychological dimensions (Goerlich-Dobre et al., 2014, 2015a; Van der Velde et al., 2014; Bermond et al., 2015). One dimension seems to correspond to a cognitive deficit related to the difficulty in identifying, analyzing, and verbalizing feelings whereas the other appears to correspond to an affective deficit based on a poor emotional and imaginative capacity to react to events able to induce emotions and activation (Vorst and Bermond, 2001; Bailey and Henry, 2007; Bermond et al., 2010). This approach was recently reinforced by findings suggesting that both dimensions could be subtended by distinct neuroanatomical profiles (Goerlich-Dobre et al., 2014, 2015b; Van der Velde et al., 2014). These profiles have been related to a disability on both dimensions, or to an exclusive disability on the affective or on the cognitive dimension. Thus, disabilities including both dimensions are mainly characterized by a reduction of gray matter in amygdala and thalamus. Cognitive disability has similar characteristics in the same regions plus insula and hippocampic areas whereas affective disability is characterized by a reduced volume of medial and anterior cingulated cortices (Van der Velde et al., 2014; Goerlich-Dobre et al., 2015a). In brief, these studies suggest that different brain processes occur in cognitive and affective alexithymia.

To explain alexithymic difficulties to deal with emotions and their consequences in social exchanges, some authors proposed that such difficulties might be related to alterations in autonomic nervous system (ANS), an important brain-body interface for interoceptive and exteroceptive exchanges, also recognized to have a central role in emotional coding of social expressions (Stone and Nielson, 2001; Franz et al., 2003; Bermond et al., 2010). In the same vein, autonomic responses have been used as robust indices of emotional brain processing (Sequeira et al., 2009; D’Hondt et al., 2010) and as markers of alexithymic reactivity (Stone and Nielson, 2001; Connelly and Denney, 2007; Bausch et al., 2011; Pollatos et al., 2011; Eastabrook et al., 2013; Constantinou et al., 2014). However, autonomic responses to emotional stimulation in alexithymia were in favor of either an hyper-reactivity (e.g., Stone and Nielson, 2001; Eastabrook et al., 2013) or an hypo-reactivity of the ANS (Franz et al., 2003; Neumann et al., 2004; Connelly and Denney, 2007; Bausch et al., 2011; Pollatos et al., 2011; Constantinou et al., 2014). While higher values of heart rate (Stone and Nielson, 2001; Eastabrook et al., 2013) and skin conductance responses (SCRs) to emotional stimulus (Stone and Nielson, 2001) were recorded in the first case, the same indices were unchanged or even attenuated in the second case. One possible explanation of these discrepancies could be that, despite the evidence in favor of the existence of cognitive and affective dimensions of alexithymia, most of the authors interested in autonomic indices did not take them into account. To our knowledge, only one study, which included female participants only, did consider alexithymic dimensions and reported higher electrodermal amplitudes in affective than in cognitive alexithymic participants (Bermond et al., 2010).

Another important factor to take into account is the social relevance of the stimuli. Previous studies reported significant effects of the social content (human vs. non-human) of emotional stimuli on the reactivity at central level, through evoked potential analysis (Proverbio et al., 2009; Groen et al., 2013) and at peripheral level, through autonomic measures (Althaus et al., 2014; Singleton et al., 2014). This social effect has been related to an automatic attentional affective processing, which facilitates the empathy (Proverbio et al., 2009; Groen et al., 2013) or social abilities (Althaus et al., 2014; Singleton et al., 2014). In this line, Singleton et al. (2014), found higher values of SCRs for social relevant stimulus than for those without social relevance, in healthy participants scoring high in autistic symptoms scale. Therefore, the distinct profiles of alexithymic dimensions characterized by neuroanatomical differences in the brain limbic structures (Van der Velde et al., 2014; Goerlich-Dobre et al., 2015a) could present different automatic attentional affective processing to social and no social stimulus, which are relevant to empathy and social processes (Proverbio et al., 2009; Bogdanov et al., 2013; Groen et al., 2013). Considering that the orientation to affective social information is fundamental for experiencing affective empathy, it is important to use a robust autonomic index of arousal related to both the cognitive and affective dimensions. In fact, SCRs are related to the activity of eccrine sweat glands exclusively innervated by the sympathetic branch of the ANS and are sensitive to variations of central activation linked to attention, preparation to the action, decision making, novelty and especially emotion, even when induced by subliminal conditions (Silvert et al., 2004; Sequeira et al., 2009; Boucsein, 2012). Thus, it appears relevant to use SCRs as a potential tool to evaluate the impact of emotional and social stimuli properties on the dimensions of alexithymia.

Overall, and considering that most reported results had been obtained in participants on the basis of the sole cognitive dimension of alexithymia, we hypothesized that behavioral and physiological inconsistencies regarding emotional and social related stimulations in alexithymia are due to an insufficient discrimination of cognitive and affective dimensions. Consequently, this research aimed to compare participants without alexithymia (NA) and with affective (AA) or cognitive alexithymia (CA) in their autonomic reactivity while viewing emotional images with and without social relevance. To this end, we recorded SCRs as a marker of the autonomic reactivity to the arousing value of the emotional images. Considering that the social and emotional deficits have been linked with alexithymia (Fukunishi et al., 1997; Moriguchi et al., 2006, 2007; Grynberg et al., 2010; Neumann et al., 2014; Hoffmann et al., 2016), we also assessed empathy, anxiety, depression, and social phobia, although the specific link between these dimensions and electrodermal variations remains a topic of debate (see Boucsein, 2012).

## Materials and Methods

The study was approved by the Ethics Committee of University of Lille 3 [Decision n° 215-6-S29], all participants gave written informed consent in accordance with the Declaration of Helsinki and received a compensation of €20.

### Participants

Demographic data and alexithymia scores of the three groups are shown in the **Table 1**. The groups did not significantly differ regarding the mean age [*F*(2,46) = 2,17; *p* = 0.126] nor the sex ratio [*X*^2^(2) = 0.35; *p* = 0.831].

**Table 1:** Demographic data and alexithymia scores of the three groups: without alexithymia (NA), and with affective (AA) and cognitive alexithymia (CA).

	NA	AA	CA	Between group differences
	***n* = 16**	***n* = 14**	***n* = 19**	
	***m* ± *s***	***m* ± *s***	***m* ± *s***	
Age	22.4 ± 2.7	22.1 ± 1.8	21.0 ± 1.6	NS
Men/Women	6/10	6/8	9/10	NS
TAS-20	38.7 ± 6.9	42.5 ± 7.2	61.1 ± 5.2	CA > (AA ∼ NA)
A-BVAQ	28.8 ± 4.4	50.7 ± 7.5	33.8 ± 7.7	AA > (CA ∼ NA)
C-BVAQ	43.1 ± 7.9	51.6 ± 6.2	77.6 ± 7.5	CA > AA > NA

*∼: NS; >: *p* < 0.05; s = standard deviation.*

Forty-nine (21 males) healthy participants aged 19–27 years were recruited for this study. None suffered from any psychiatric or neurological disorder according to self-report. They had to be French speakers and to present normal or corrected-to-normal vision. Participants were distributed into the NA, AA, and CA groups according to their scores to the TAS-20 and the affective (A-BVAQ) and cognitive (C-BVAQ) sub-scales of BVAQ (Bagby et al., 1994; Loas et al., 2001; Bailey and Henry, 2007). For the group without alexithymia (NA), the inclusion criteria were: TAS ≤ 44, A-BVAQ ≤ 44, and C-BVAQ ≤ 64. The affective (AA) and cognitive (CA) groups were distinguished on the basis of BVAQ sub-scales: A-BVAQ > 44 and C-BVAQ ≤ 64 for the AA group, and A-BVAQ ≤ 44 and C-BVAQ > 64 for CA group. As expected, TAS-20 and C-BVAQ scores correlated [*r*(47) = 0.88; *p* < 0.001] while A-BVAQ scores did not correlate with any of them [*p*s > 0.450]. The internal consistency of alexithymia scales was assessed on the whole selection of 49 participants using the Cronbach’α coefficient. It proved to be very good for both the TAS (α = 0.84) and the BVAQ (0.86), as well as for the affective (0.85) and the cognitive (0.92) subscores of the BVAQ.

The TAS-20 (Bagby et al., 1994; Taylor et al., 2003) is a self-questionnaire which comprises 20 items distributed in three sub-scales: difficulty to identify feelings, difficulty to describe feelings, and the presence of externally oriented thinking. The participants completed each item on a 5-point scale from ‘strongly disagree’ to ‘strongly agree’ (Bagby et al., 1994; Taylor et al., 2003). The BVAQ was also applied in order to distinguish between affective and cognitive dimensions of alexithymia (Vorst and Bermond, 2001). The BVAQ is a self-report questionnaire that includes 40-items in five sub-scales, three related to the cognitive dimension (C-BVAQ: “Verbalizing,” “Identifying,” and “Analyzing emotions”) and two related to an affective dimension (A-BVAQ: “Emotionalizing” and “Fantasizing”). Both TAS-20 and BVAQ have been validated in French population (Taylor et al., 2003; Deborde et al., 2008) and both proved to have high validity to assess alexithymia (Bermond et al., 2015). The validity of the two-factor structure of BVAQ has been confirmed through factor analyses (Bailey and Henry, 2007; Bermond et al., 2007; but see Bagby et al., 2009 for failure to support the two-factor structure). The correlation between the cognitive BVAQ factor and the total score of the TAS-20 is high (*r* = 0.80), indicating that they assess the same alexithymic features (Vorst and Bermond, 2001).

### Empathy and Clinical Questionnaires

Empathy, as well as some clinical (social phobia, anxiety, and depression) characteristics of participants were evaluated in order to study their relationship with alexithymia (Grynberg et al., 2010). These characteristics were measured by self-administered questionnaires: the Social Phobia Scale (Liebowitz, 1987), the State-Trait Anxiety Inventory (STAI), with includes the STAI-A for state (Spielberger et al., 1970) and STAI-B for trait anxiety (Spielberger et al., 1993), and the Beck Depression Inventory (BDI-II, Beck et al., 1996). The components of empathy were evaluated through the Interpersonal Reactivity Index (IRI, Davis, 1983), which is one of the most widely used self-report questionnaires due to its multidimensional ability to approach the empathy. The inventory includes four sub-scales: Perspective Taking (PT), Fantasy (FS), Personal Distress (PD), and Empathic Concern (EC). A suggested dichotomy of these components considered that the PT sub-scale evaluates the cognitive empathy while the other three sub-scales assess the affective empathy (De Corte et al., 2007).

### Stimuli

We selected 120 pictures (1024 × 768 pixels) from the International Affective Picture System (IAPS; Lang et al., 2008) distributed in two series differing by their social relevance, i.e., the social content of the pictures (SCP): the pictures of the social series (SO) showed one or more human beings, while that of the non-social series (NSO) showed animals or inanimate objects. This selection was similar to that of Proverbio et al. (2009) or Groen et al. (2013). Each series included 60 pictures distributed in three sets (3 × 20) differing by the emotional content of the pictures (ECP): unpleasant (U), neutral (N), and pleasant (P). The IAPS arousal scores of the selected pictures showed the expected arousal effect [*F*(1,114) = 203.99; *p* < 0.001]: the mean scores of U and P sets were 63% higher than those of N sets. This quadratic contrast explained 99% of the variance linked to ECP factor and did not depend on SCP [*F*(1,114) = 0.24; *p* = 0.623].

### Skin Conductance Recording

Skin conductance responses were recorded using a Biopac MP150 system with a sampling frequency of 500 Hz. The signal was captured by bipolar placement of Ag/AgCl standard surface electrodes filled with an isotonic electrolyte paste on the index and middle fingers of the non-dominant hand of the participant. The conductance was measured with a gain of 5 μΩ/V and a low-pass filter of 10 Hz. The raw signal was calibrated to detect activity in the 0–100 μSiemens range. Data were collected with the AcqKnowledge software (Biopac) on a computer different from that which controlled the task. Any elicited response appearing 1 s after the onset of the first picture of each set and having a superior or equal to 0.05 μS amplitude was considered as a SCR (Venables and Christie, 1980). Thus, the number of SCRs and their mean amplitude were calculated for each set of pictures through the LabChart software.

### Procedure and Task

Before the experiment, participants fulfilled the TAS-20 and the BVAQ in order to evaluate the alexithymic components of each participant and to include her/him in one of the three groups. The individuals who met the aforementioned criteria were invited to participate in the experiment, at the IrDive Platform Imaginarium (Tourcoing, France). The whole experiment was conducted individually. First, the participants responded the clinical and empathy questionnaires. Thereafter, in order to obtain a stabilized electrodermal recording, the electrodes were attached 5 min before the beginning of the task during which participants passively viewed the images.

The size of the pictures on the computer screen was 55 cm and the distance between the participant and the screen was 77 cm. The presentation of the pictures was organized in two blocks separated by a 5-min break: one corresponded to the SO series and the other to the NSO series. The order of the blocks was counterbalanced across the participants. For both blocks, U, N, and P sets followed each other and were counterbalanced across participants. Each set lasted 1 min 50 s and was separated from the next one by a 2-min delay, necessary to recover the electrodermal rest activity. The presentation of each picture lasted 4 s and was followed by an “^∗^” that lasted for 500 ms, then the screen remained black for 500 ms and, finally, an arrow, oriented to the left (<) or the right (>) was presented during 500 ms. After the visualization of each picture, and in order to keep the participant’s gaze fixed on the screen, the participant had to respond by a click on the computer mouse according to the direction of the arrow. The SCR was recorded during the whole duration of both blocks. At the end of the task, the participant was asked to look again at all pictures and to rate the arousal value using a 9-point SAM scale (Self Assessment Manikin; Bradley and Lang, 1994): from 1, very calm, to 9, very arousing).

### Data Analysis

The analyses of age and psychometric scores (TAS-20, A-BVQA, C-BVAQ, BDI, STAI-A, STAI-B, IRI sub-scales) were carried out by comparing the groups (NA, AA, CA) means with a one-way ANOVA and *post hoc* Bonferroni tests. Subjective arousal and the number and the mean amplitude of SCRs were analyzed according to a three way repeated-measures design taking into account the factors ECP (U, N, P), SCP (SO, NSO), and group (NA, AA, CA). Planned quadratic contrasts (QC: U/2+P/2-N) were used in order to extract the arousal emotional effect from the global emotional effect (see D’Hondt et al., 2010). The correlations between the variables were tested with the coefficient of Pearson (*r*). An effect was considered significant when *p* was ≤ 0.05.

## Results

### Empathy and Clinical Measures

The **Table 2** summarizes the empathy and clinical measures obtained in the three groups.

**Table 2:** Empathy and clinical scores of the three groups: without alexithymia (NA), and with affective (AA) and cognitive alexithymia (CA).

	NA	AA	CA	α	Between groups differences
	***n* = 16**	***n* = 14**	***n* = 19**	***n* = 49**	
	***m* ± *s***	***m* ± *s***	***m* ± *s***		
C-IRI/PT	39.9 ± 3.6	36.5 ± 3.5	32.5 ± 7.4	0.69	NA > CA
A-IRI	102.3 ± 14.8	85.2 ± 15.6	99.9 ± 17.1	0.84	(NA ~ CA) > AA
A-IRI/FS	38.6 ± 6.6	29.5 ± 6.9	34.8 ± 9.0	0.79	NA > AA
A-IRI/PD	24.4 ± 6.7	19.4 ± 5.1	28.3 ± 8.9	0.81	CA > AA
A-IRI/EC	39.3 ± 6.7	36.4 ± 9.2	36.8 ± 8.0	0.86	NS
Social phobia	36.3 ± 15.8	36.6 ± 18.2	53.7 ± 21.9	0.94	CA > (AA ~ NA)
STAI-A	28.6 ± 5.6	29.6 ± 5.0	36.1 ± 10.1	0.91	CA > (AA ~ NA)
STAI-B	37 ± 6.9	34.4 ± 3.6	47.5 ± 12.1	0.92	CA > (NA ~ AA)
BDI	6.9 ± 6.6	5.1 ± 2.8	13.5 ± 9.4	0.90	CA > (NA ~ AA)

*~: NS; >: *p* < 0.05.*

*Cronbach’s alpha (α) reached 0.69 for PT sub-scale and was fairly high in all other cases.*

Regarding the empathy scale (IRI), the cognitive sub-score (PT sub-scale) [*F*(2,46) = 8.39, η^2^ = 0.27, *p* < 0.001] differentiated CA and NA (*p* = 0.001) groups. The affective sub-score [*F*(2,46) = 4.99, η^2^ = 0.18, *p* < 0.011] distinguished the AA group: NA and CA group did not differ (*p* = 1.000) and both had greater scores than AA (*p*s < 0.036). More detailed analyses showed that the scores were greater than those of AA group for NA group on FS sub-scale (*p* = 0.007) and for CA group on PD sub-scale (*p* = 0.003).

Social phobia varied according to the groups [*F*(2,46) = 4.75, η^2^ = 0.17, *p* = 0.013], with higher scores obtained by CA group than NA (*p* = 0.03) and AA groups (*p* = 0.05). On STAI-A scale [*F*(2,46) = 5.05, η^2^ = 0.18, *p* = 0.010], the CA group scored higher than NA (*p* = 0.017) and AA groups (*p* = 0.058). The same was true for STAI-B [*F*(2,46) = 10.94, η^2^ = 0.32, *p* < 0.001]: the mean score was higher for CA group than for NA (*p* < 0.003) and AA groups (*p* < 0.001). On the depression scale (BDI) [*F*(2,46) = 6.47, η^2^ = 0.22, *p* = 0.003], the score of CA group was also higher than that of NA (*p* = 0.030) and AA (*p* = 0.005). AA and NA groups did not differ on these three scales (*p* = 1.00 in each case).

### Subjective Arousing Value of the Pictures

The mean arousal judgments of the pictures did not differ according to the groups [*F*(2,46) = 0.70, η^2^ = 0.03, *p* = 0.501]. Moreover, ECP affected in the same way the arousal judgments of the three groups [*F*(4,92) = 1.62, η^2^ = 0.07, *p* = 0.174]. As expected the scores were higher (by 64%) for emotional pictures than for neutral ones. This arousal contrast [QC, *F*(1,46) = 203.3, η^2^ = 0.76, *p* < 0.001] explained 64% of the variance linked to ECP factor. SCP modulated [*F*(1,46) = 10.63, η^2^ = 0.19, *p* = 0.002] the mean arousal judgments similarly in the three groups [*F*(2,46) = 2.38, η^2^ = 0.09, *p* = 0.103]. There was no interaction effect between ECP and SCP factors [*F*(2,92) = 0.78, η^2^ = 0.02, *p* = 0.459], whatever the group [*F*(4,92) = 1.09, η^2^ = 0.04, *p* = 0.364].

### Skin Conductance Responses

**FIGURE 1:**
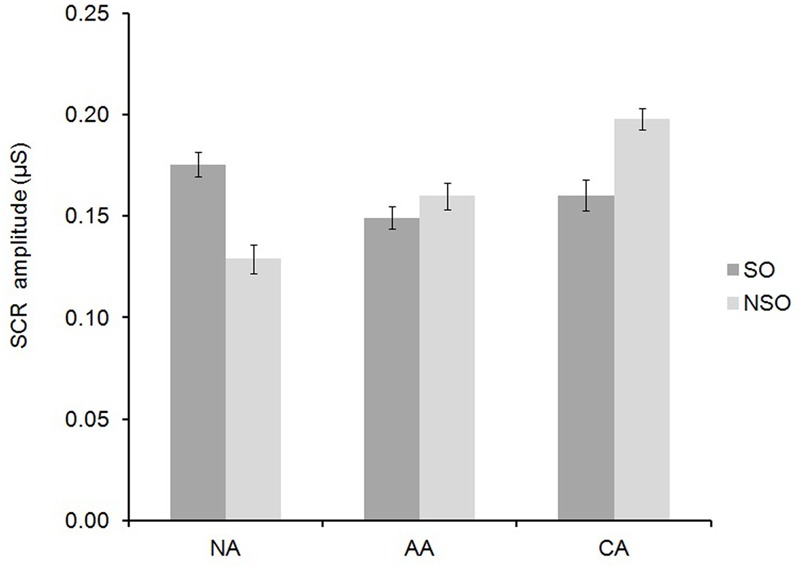
**Amplitude of skin conductance responses (SCRs) to pictures with social (SO) and without social relevance (NSO) in each group: without alexithymia (NA), and with affective (AA) or cognitive alexithymia (CA).** Group means ± standard error on the means.

Emotional content of the pictures tended to modulate the SCR amplitude [*F*(2,92) = 2.50, η^2^ = 0.05, *p* = 0.087], in the same way in the three groups [*F*(4,92) = 0.80, η^2^ = 0.03, *p* = 0.528]: a contrast analysis revealed that this was due to an activation effect (QC:, η^2^ = 0.07, *p* = 0.06), the emotional pictures increasing SCRs amplitude by 15% as compared to neutral ones. SCP tended to influence the rate of SCRs [*F*(1,46) = 3.74, η^2^ = 0.07, *p* = 0.059], similarly in the three groups [*F*(2,46) = 1.98, η^2^ = 0.08, *p* = 0.149]: 10% more responses occurred when the pictures contained humans. However, the effect of SCP on SCR amplitude was significantly different according to the group [*F*(2,46) = 4.29, η^2^ = 0.16, *p* = 0.019, **Figure 1**]. A contrast analysis revealed that, compared to the higher amplitude for human content shown by non-alexithymic group [(SO-NSO)_NA_; *p* = 0.034], the difference tended to disappear in affective alexithymic group [(SO-NSO)_NA_ - (SO-NSO)_AA_; *p* = 0.06] and did reverse in cognitive alexithymic group [(SO-NSO)_NA_ - (SO-NSO)_CA_; *p* < 0.01]. The interactions between ECP and SCP, and between ECP, SCP, and group did not reach significance, neither on SCR rate nor on SCR amplitude (η^2^ ≤ 0.06, *p* > 0.204 in each case).

### Correlations

In the NA group, the effect of SCP on SCR rate positively correlated with its effect on activation judgments [*r*(14) = 0.56; *p* = 0.025]. This correlation vanished in AA and CA groups (|*r*| < 0.15 in each case).

The effects of SCP on SCR parameters did not correlate with the scores on social phobia scale, STAI-A and STAI-B whatever the group. The only exception concerned the BDI which negatively correlated with the SCP effect on SCR rate in NA [*r*(14) = -0.56; *p* = 0.025] and AA groups [*r*(12) = -0.54; *p* = 0.047].

## Discussion

The aim of this study was to compare the effects of emotional and social visual stimulations on SCRs in participants differing along the affective and cognitive dimensions of alexithymia. Firstly, the data confirmed several classical effects: cognitive alexithymics were more anxious and depressive and presented higher scores of social phobia than non-alexithymics; human pictures induced more marked emotional judgments compared to non-human pictures; the amplitude of SCRs tended to reflect the arousing value of the emotional stimuli. Secondly, this experiment has two original outcomes: affective alexithymics expressed lower empathic affective scores than other groups; alexithymia modulated the impact of the social relevance of the stimuli on the autonomic reactivity. Interestingly, this impact vanished in affective alexithymics and reversed in cognitive alexithymics. Thus, though the groups could not be distinguished on the basis of emotional effect on SCRs, NA and CA groups clearly differed when the impact of social relevance was considered.

### Affective vs. Cognitive Alexithymic Profiles

Concerning the clinical features, the CA group distinguished itself from both other groups by higher social phobia, anxiety (trait and state), and depression. In line with these data, several previous studies evaluating the cognitive alexithymia through TAS-20 (Bagby et al., 1994) also found a link with these features: depression (Hesse and Floyd, 2008; Grynberg et al., 2010, 2012), anxiety (Grynberg et al., 2010, 2012) or social phobia (Fukunishi et al., 1997). The fact that the AA group did not differ from the NA group on these scales constitutes an argument in favor of the bidimensional conception of alexithymia separating a cognitive dimension from an affective one. Besides, the CA group obtained lower scores than NA on the PT sub-scale of empathy inventory. This poor capacity to adopt the point of view of others is consistent with the idea that this group has main difficulties at the cognitive level (Vorst and Bermond, 2001). Some studies based on the TAS-20, i.e., an evaluation that makes no difference between affective and cognitive dimensions, reported lower scores in PT factor in alexithymia (Moriguchi et al., 2006, 2007; Neumann et al., 2014; Hoffmann et al., 2016), but also on other sub-scales constituting the affective part of the empathy inventory, such as PD (Moriguchi et al., 2007) or FS (Grynberg et al., 2010).

In fact, our bidimensional approach using the BVAQ revealed that only the AA group distinguished itself from both others on the global affective empathy sub-score. Again, the fact that the CA group did not differ from NA group on this sub-score is in favor of the bidimensional conception of alexithymia. More detailed analyses revealed that the AA group scored lower than NA on FS factor, suggesting a lower capacity of fantasy, e.g., a poor skill to imagine themselves inside stories or movies (Davis, 1983). The affective dimension of alexithymia could mainly concern this aspect according to Vorst and Bermond (2001). On the PD sub-scale, AA group had lower scores than CA. This sub-scale is thought to assess the vulnerability to stress or to contagion when facing uncomfortable feelings like anxiety (Davis, 1983; Hoffmann et al., 2016). A recent study evaluating alexithymia with TAS-20, reported a link between higher PD scores and depression, and suggested that the tendency to emotional contagion commonly associated with depression can be attributed to this factor (Hoffmann et al., 2016). The fact that the AA and NA groups showed a negative correlation between the effect of SCP on SCR and the BDI scores on one hand and the non-existence of such correlation for CA participants on the other hand constitutes another argument in favor of the bidimensional conception of alexithymia separating a cognitive dimension from an affective one.

In brief, the use of complementary tools to evaluate alexithymia allowed a sound clinical characterization of alexithymics and their individualization in terms of affective or cognitive orientation. This may provide relevant information which could be useful in order to improve therapeutical treatments: i.e., an evaluation of emotional disorders such as anxiety and depression as well as of the perspective taking ability could benefit to cognitive alexithymics whereas special attention could be paid to empathic abilities in the case of affective alexithymics. Furthermore, considering the interest of neurofeedback to gain control over autonomic responses, electrodermal neurofeedback could be used to change autonomic arousal related to social component of visual stimulations in alexithymics, as revealed by SCRs amplitude.

### Effect of Emotion

The subjective evaluation of arousal value of emotional stimuli did not depend on the groups. This result is quite surprising insofar as alexithymics individuals are characterized by difficulties in identifying and describing their emotions, probably related to the subjective sensitivity of internal bodily sensations (Betka et al., personal communication). In this frame, it could be hypothesized that the emotional power of pictures was not strong enough to induce specific components of interoceptive sensibility leading to differential subjective judgements in alexithymic individuals. However, the evaluation of arousal was modulated by the social content of pictures, the emotional judgments being systematically more marked for pictures having a human content when compared to those showing a non-human content.

As expected, the autonomic reactivity was modulated by the emotional content of the pictures. An arousal effect is supported by the fact that the SCR amplitude tended to be higher with emotional than with neutral stimulation which is consistent with the literature (Bradley and Lang, 2000; Khalfa et al., 2002; Sequeira et al., 2009; D’Hondt et al., 2010). In the current study, the arousal effect was rather weak which could result from the blocked procedure we used for presenting the different sets of emotional pictures. Varying the emotional value from one picture to another seems more typical of the studies which showed strong arousal effects (Bradley and Lang, 2000; Sequeira et al., 2009; D’Hondt et al., 2010). The relative weakness of the emotional effect on SCRs could explain why no group difference emerged in the present study, contrary to that reported by Bermond et al. (2010). These authors showed a higher arousal effect with the affective than with the cognitive dimension of alexithymia. However, as they recognized, their emotion-inducing situation was limited to fear and erotic pictures and needed to be generalized to various emotion inductions, as carried out in this work. Another possible explanation could be the gender effect, because in the present study we included both sexes, while Bermond et al. (2010) only evaluated females. More studies are necessary to explore the possible sexual differences in cognitive and affective dimensions of alexithymia.

### Effect of Social Relevance

Though the social and NSO series of pictures were presented by block too, we obtained a clear effect on SCRs. The hyper-reactivity to socially relevant stimuli recorded in NA group, vanished in AA and reversed in favor of non-socially relevant stimuli in CA group. The low autonomic reactivity observed in CA group to social relevant information was not linked to values of social phobia, anxiety, and depression. Previous studies on social relevance showed significant differences on the reactivity at central (Proverbio et al., 2009; Groen et al., 2013) and peripheral levels (Althaus et al., 2014; Singleton et al., 2014). Regarding peripheral level and particularly skin conductance measures, it has been reported that healthy participants showed higher SCRs to stimulus with than without social relevance, while subjects with some social disorders (e.g., autism) showed the opposite pattern (Singleton et al., 2014). In the same way, we found an inversion between NA and CA participants. We have to remember that if the SCR has been widely related with the activation originating in emotions (Bradley and Lang, 2000; Critchley, 2002; Sequeira et al., 2009), it has also been associated with attention and motivational significance of the stimulus (Bradley, 2009; Singleton et al., 2014). In this line, the SCR is considered as an orienting response, in accordance with the role of the sympathetic system in arousing and engaging behavioral and physiological reactions to salient stimulation (Bradley, 2009; Sequeira et al., 2009; Boucsein, 2012). Indeed, the higher electrodermal reactivity shown in the current study may reflect the interest of non-alexithymic participants for social stimuli which can be related with data obtained in typically developing adolescents (Louwerse et al., 2014). Interestingly, the AA group did not show any difference between stimulus with or without social relevance. This suggests that AA participants experienced the two types of scenes as equally relevant which could be related to their low scores in affective empathic scales. Additionally, our results cannot be attributed to differences on perception of stimulus since the groups did not differ as for their subjective assessments of pictures.

Finally, the hyper-reactivity to socially relevant stimuli in NA group was replaced by a hypo-reactivity to the same stimuli in CA participants. Such reactivity is in line with the hypo-arousal model of alexithymia proposed by Linden et al. (1996). Though the literature regarding the links between autonomic variables and alexithymia has produced equivocal results, the current study as that of Bermond et al. (2010) presents the advantage to separately evaluate affective and cognitive dimensions of alexithymia. Thus, considering on one side the sensitivity of the skin conductance activity to emotional, attentional or other arousing stimuli, and on the other side the difficulty for CA participants to verbalize, identify, and analyze emotions, a low autonomic reactivity to socially relevant pictures is quite coherent. Furthermore, the low scores of CA obtained in the PT sub-scale of empathy inventory, showing a difficulty to adopt the point of view of others, could be related with the incapacity of CA participants to experience the activation value usually associated to social content of pictures and thus contribute to explain the low level of autonomic reactivity. This low capacity to mobilize the autonomic sphere is in line with the idea that alexithymia could be secondary to a failure to centrally integrate a low flow of bodily signals (Brewer et al., 2015). In this context, the low level of autonomic activation to socially relevant stimuli could bear testimony of an insufficient contribution of interoception to guide social interactions on the basis of verbalizing and identifying emotions (Garfinkel et al., 2015).

## Conclusion

This study is the first to suggest a specific autonomic reactivity to socially relevant standardized stimuli in cognitive alexithymia. The value of this result is specially reinforced by the joint utilization of the classical TAS-20 and the BVAQ self-report questionnaires, a double psychometric procedure allowing a robust clinical characterization of affective and cognitive alexithymia. Furthermore, the same result cannot be attributed to differences on perception of stimulus since the groups did not differ as for their subjective assessments of pictures. Another interest of this study corresponds to the fact that, as proposed by Bermond et al. (2010), our experiment extends the analysis to the use of a large variety of standardized emotional pictures, unpleasant and pleasant.

In spite of the originality and the interest of this work, we have to recognize some limitations. First, the presentation of stimuli in blocked sets associated with a secondary task to indicate the orientation of an arrow could have produced some non-specific SCRs which impact needs to be evaluated. In this perspective, the time between each stimulation could be increased in order to avoid interactions between SCRs elicited by individual pictures. Secondly, the dichotomy adopted to distinguish cognitive and affective components of empathy remains a matter of discussion in the literature regarding FS sub-scale, which some authors rather consider as a cognitive component of empathy (Cusi et al., 2011; Dziobek et al., 2011). Thirdly, although this study integrated females and males, the size of the sample was not great enough to extract possible gender differences. This point needs to be deeply assessed in future studies. In a more prospective way, the analysis of the autonomic impact of alexithymia would be enriched by the analysis of parasympathetic branch, a major vehicle of interoceptive signals able to interact with subjective components of alexithymia. Another important issue is related to the distinction human/non-human vs. social/non-social. Though our approach was similar to that of other authors (Proverbio et al., 2009; Groen et al., 2013; Singleton et al., 2014), it could be of interest to disentangle in future designs two aspects contained in what was called ‘social’ factor, by using for example pictures showing one vs. several individuals belonging to human vs. non-human species. In addition, considering that strategies of each group to perceive and analyze social vs. non-social emotional stimuli need to be better understood, we are running experiments integrating eye saccadic movements during the projection of each picture to explore this issue. A last point concerns the individuals scoring high both on affective and cognitive BVAQ, not explored in the present work. One may wonder whether further studies of affective and cognitive alexithymia could benefit from the inclusion of such a group.

Finally, on the basis of well differentiated groups in terms of alexithymia, this study brings data about a specific autonomic signature able to contribute to elucidate the difficulty of alexithymic participants to integrate interoceptive information in order to regulate their social transactions when based on arousing information. The present research provides an original track toward psychophysiological foundations of alexithymia according to affective and cognitive dimensions, and it is the first study to show differences between social and non-social stimulus on SCR depending on alexithymia dimensions.

## Authors Contributions

EM-V contributed to the study on conception and design, the acquisition, the analysis of data and the manuscript writing; JH performed the statistical analysis and contributed to the manuscript writing and the interpretation of results; LdZ, helped to the acquisition and the analysis of data; JR-L reviewed the manuscript and provided critical revisions; HS contributed to the study on conception and design, interpretation of data and supervised the manuscript writing. All authors approved the final version of the manuscript for submission.

## Conflict of Interest Statement

The authors declare that the research was conducted in the absence of any commercial or financial relationships that could be construed as a potential conflict of interest.
